# Physiologic Functional Evaluation of Left Internal Mammary Artery Graft to Left Anterior Descending Coronary Artery Steal due to Unligated First Thoracic Branch in a Case of Refractory Angina

**DOI:** 10.1155/2016/3175798

**Published:** 2016-02-14

**Authors:** Fadi J. Sawaya, Henry Liberman, Chandan Devireddy

**Affiliations:** Department of Medicine, Division of Cardiology, Emory University School of Medicine, Atlanta, GA 30308, USA

## Abstract

Unligated side branches of the left internal mammary artery (LIMA) have been described in the literature as a cause of coronary steal resulting in angina. Despite a number of studies reporting successful side branch embolization to relieve symptoms, this phenomenon remains controversial. Hemodynamic evidence of coronary steal using angiographic and intravascular Doppler techniques has been supported by some and rejected by others. In this case study using an intracoronary Doppler wire with adenosine, we demonstrate that a trial occlusion of the LIMA thoracic side branch with selective balloon inflation can confirm physiologic significant steal and whether coil embolization of the side branch is indicated.

## 1. Introduction

The internal mammary artery is the graft of choice in coronary artery bypass (CABG) surgery given its favorable long-term 90% patency at 10 years compared to saphenous vein grafts (SVG) [1, 2]. Left internal mammary artery (LIMA) side branches to the chest wall have been reported to occur in 10 to 20% of patients in preoperative and postoperative data [3]. Preferential blood flow through these unligated thoracic branches and subsequent coronary steal phenomena have been reported as potential causes of angina [4]. Successful ligation of these side branches surgically or through catheter embolization has been documented in the literature to effectively relieve anginal symptoms through mainly subjective measures [5, 6]. However, the hemodynamic significance of large thoracic side branches has been largely debated and is still controversial [7]. We report here a case of refractory angina in a patient with history of CABG surgery where we physiologically demonstrate coronary steal via a large unligated thoracic side branch by measuring coronary flow reserve before and after selective thoracic side branch balloon occlusion and successful treatment by coil embolization of the branch.

## 2. Case Report

**Figure 1:**
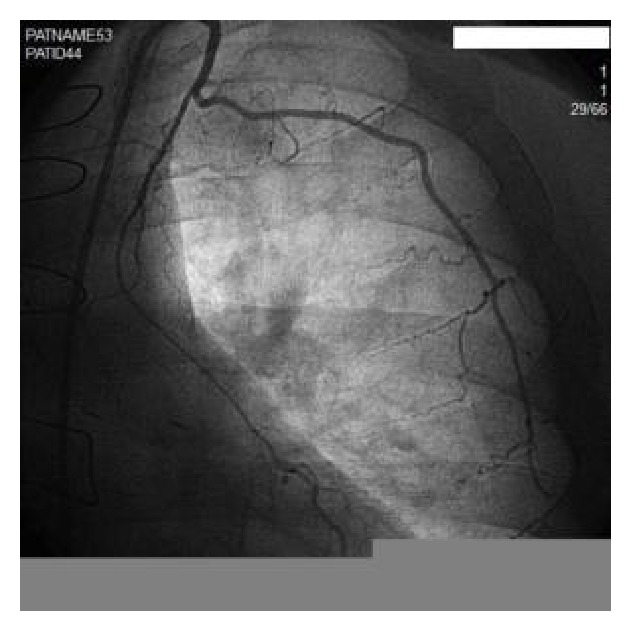
Patent hypoplastic LIMA with large thoracic side branch.

A 50-year-old male with known history of coronary artery disease (CAD) and history of 2 vessel CABG 10 years previously presented to our facility with unstable angina. Over the preceding months, the patient experienced Canadian classification class III exertional angina despite maximal medical therapy with beta-blockers, calcium-channel blockers, and long acting nitrates. Given his worsening angina, a diagnostic catheterization was performed showing luminal irregularities in his circumflex artery (LCX), a 100% stenosis in his mid left anterior descending artery (LAD), and a 100% stenosis in his first diagonal artery. His right coronary artery (RCA) had a 95% stenosis in the posterolateral ventricular branch (PLV). His grafts showed a patent SVG to first diagonal and a small hypoplastic patent LIMA to LAD (Figure 1). There was no further progression of native coronary artery disease compared to prior cardiac catheterization expect for a large, laterally directed LIMA first intercostal thoracic side branch suggesting possible coronary steal and preferential blood flow through the intercostal branch. The large thoracic side branch was barely apparent on an angiogram done after his bypass surgery. The PLV branch was presumed to be the culprit lesion causing the patient's anginal symptoms and a 2.5 mm × 23 mm Promus drug eluting stent (Promus PREMIER*™*, Boston Scientific, Natick, MA) was deployed in the PLV with excellent angiographic results.

Despite coronary intervention, the patient continued to experience significant exertional angina. A cardiac positron emission tomography (PET) scan was performed demonstrating a 10–15% reversible anteroapical and anterolateral ischemic defect confirming suspicion of possible coronary steal from the LIMA thoracic side branch. In light of the PET result, the patient was brought back to the catheterization laboratory with coronary flow reserve (CFR) chosen to measure the functional significance of the thoracic branch.

**Figure 2:**
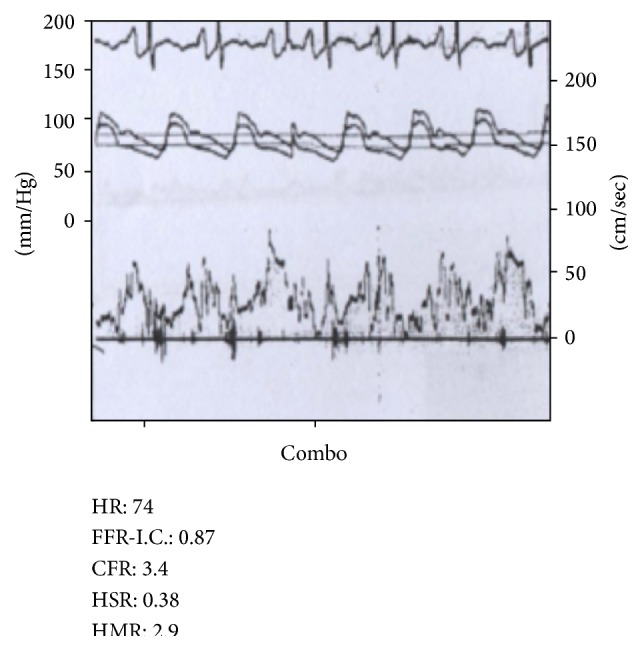
Coronary flow reserve (CFR) tracing during intravenous adenosine infusion prior to balloon directed occlusion revealing a value of 3.4 after IV adenosine 140 mcg/kg/min.

**Figure 3:**
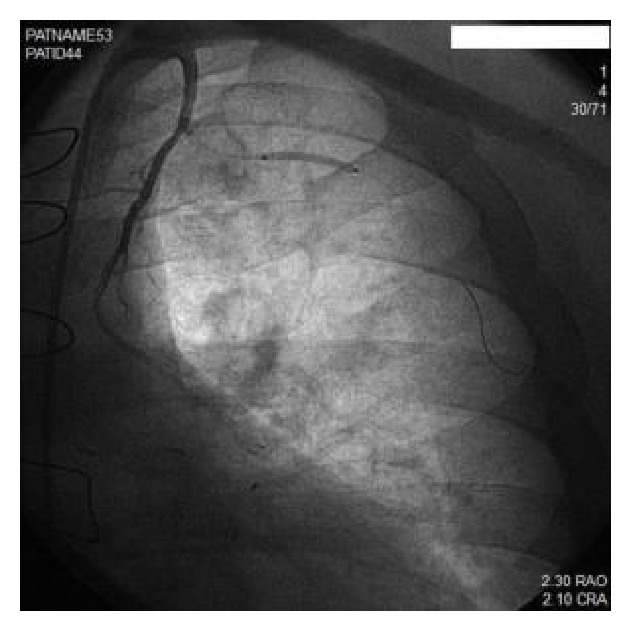
The LIMA was engaged with a 6-French IMA guide catheter. The Doppler wire was then advanced into the mid-portion of the LIMA distal to the first thoracic branch collateral with balloon occlusion of side branch.

**Figure 4:**
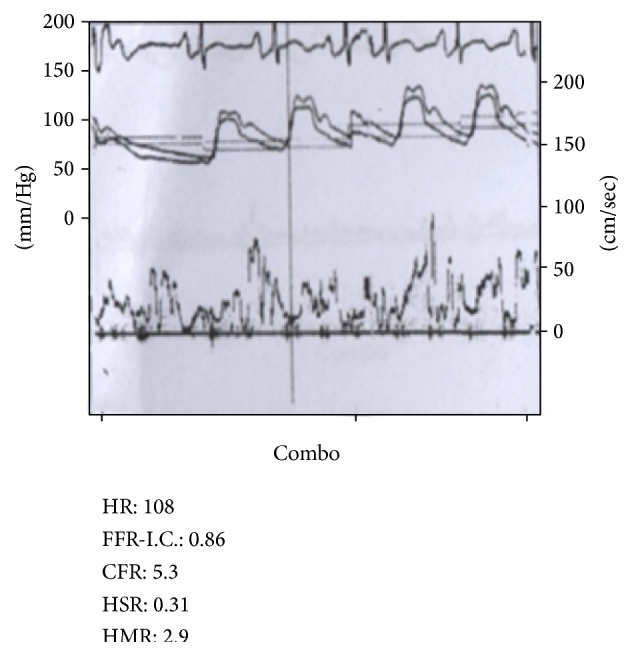
Coronary flow reserve (CFR) tracing during 140 mcg/kg/min intravenous adenosine infusion following side branch balloon occlusion revealing a value of 5.3.

**Figure 5:**
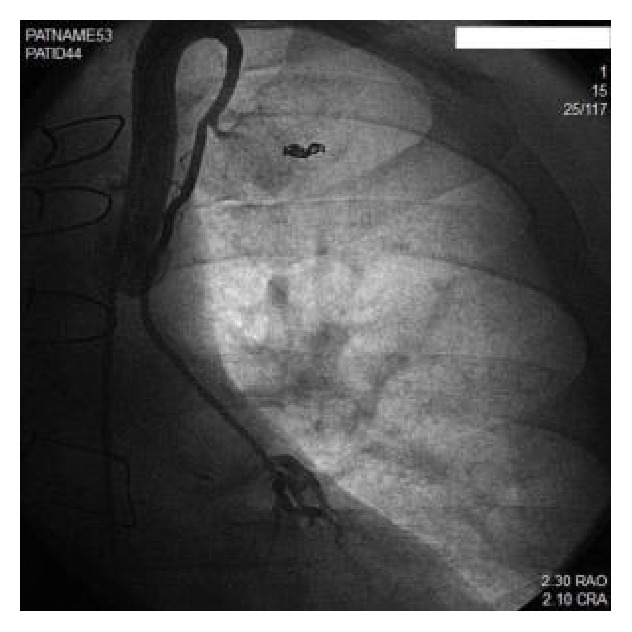
Angiogram of the LIMA showing successful microembolization of the thoracic side branch after placement of 4 microcoils with concomitant significant increase in LIMA size.

The LIMA was engaged with a 6-French IMA guiding catheter (Cordis Corporation, Miami, FL). The coronary Doppler wire (FloWire, Volcano, San Diego, CA) was advanced into the mid-portion of LIMA distal to the first thoracic branch collateral. CFR after systemic intravenous (IV) adenosine injection (140 mcg/kg/min) was measured, and a baseline value of 3.4 was recorded (Figure 2). We then advanced, in parallel with the CFR wire, an exchange length Intuition wire (Medtronic, Minneapolis, MN) into the large first intercostal side branch over which a 2.0 × 20 mm Sprinter Legend balloon (Medtronic, Minneapolis, MN) was advanced. The balloon was inflated within the thoracic side branch inducing complete occlusion of flow (Figure 3). The CFR measurement was repeated with IV adenosine infusion (140 mcg/min/kg) during thoracic side branch occlusion revealing a value of 5.3. The discrepancy in CFR measurement from the time before and after side branch occlusion was consistent with significant coronary steal through this branch from the left internal mammary artery (Figure 4). The decision was made to proceed with embolization of the thoracic branch to restore primary graft flow to the LAD territory. The CFR wire was removed and the balloon was exchanged over the Intuition wire for a Tornado delivery catheter (Cook Medical, Bloomington, IN) deep into the thoracic side branch. Through the delivery catheter, four 3 × 2 mm Cook Miraflex microcoils (Miraflex*™*, Cook Medical, Bloomington, IN) were successfully delivered using a long 0.018′′ Steelcore wire (Abbott Vascular, Santa Clara, CA) with successful closure of the side branch. Functional closure of the thoracic branch was tested by selective injection of contrast through the microdelivery catheter. Once complete embolization was documented, the microdelivery catheter was removed and final images of the LIMA demonstrated significant increase in flow and caliber of the vessel (Figure 5). At three-month follow-up, the patient reported sustained significant improvement in angina. Given his marked improvement, repeat cardiac PET to assess him for ischemia was not justified in our opinion and would have exposed him to unnecessary radiation.

## 3. Discussion

LIMA is the conduit of choice for CABG surgery given its favorable long-term patency outcomes. Large unligated thoracic branches have been documented to occur in 10 to 20% of LIMA grafts. The clinical significance of these side branches has been largely debated and the decision to routinely ligate this branch at the time of CABG or later occlude the branch in the catheterization laboratory has remained controversial.

Previous reports supporting the concept of IMA thoracic side branch steal and interventions to occlude the thoracic side branch have postulated improvement in myocardial ischemia through predominantly subjective measures of symptomatic angina relief or through a few reports of resolution of myocardial ischemia on stress testing [8–11].

Opposing viewpoints have rejected the idea of utilizing subjective measurements as an endpoint to justify LIMA thoracic branch occlusion. In fact, Kern has suggested that LIMA side branch steal is defined as a systolic flow diversion and not a true coronary flow steal because the arterial flow to the chest wall is predominantly systolic opposed to coronary flow which is predominantly diastolic [12]. In addition, large unligated side branches have been described without any evidence of clinical symptom [13, 14] and studies that used intravascular Doppler techniques have mainly refuted this syndrome. Studies performed by Luise et al. [15], Abhyankar et al. [7], Guzon et al. [16], and Kern et al. [17] have failed to show any clinical and hemodynamic significance of IMA side branches on coronary pathophysiology under hyperemic conditions using adenosine with no change in coronary flow reserve. In addition, Gaudino et al. failed to show any significant change in LIMA coronary flow under conditions that produce both peripheral and coronary vasodilatation.

On the other hand, a study by Morocutti et al. was able to reproduce our findings. Using an intracoronary Doppler wire with IV adenosine, they demonstrated that a trial of balloon occlusion of a LIMA thoracic side branch increased flow through the LIMA (CFR of 1.6 to CFR of 3.3) confirming hemodynamically significant steal. They subsequently proceeded with successful microembolization in a similar fashion to our case [18].

The pathophysiology behind why IMA branch occlusion improves coronary flow in some and not in others is not fully known and needs to be further elucidated. Using intracoronary flow reserve and Doppler velocities, a trial occlusion of the LIMA side branch via balloon inflation can easily demonstrate whether flow downstream through the LIMA would increase after the intervention and would justify the risk of undergoing coil embolization or surgical ligation of the thoracic side branch. Ligation has been performed mainly by the means of coil embolization with one group deploying a vascular plug to obstruct flow into the side branch [19].

We highly recommend that the hemodynamic functional significance of an unligated thoracic side branch of the LIMA be confirmed using coronary flow reserve measurement after balloon occlusion of the side branch. This provides objective stratification of unligated side branches based on validated measures of coronary flow and can potentially improve the quality of life for patients suffering angina as a result of thoracic side branch coronary steal.
